# A clinicopathological study on lupus nephritis; experience of 34 cases from Bangladesh

**Published:** 2015-07-22

**Authors:** Muhammad Nazmul Baqui, Shabnam Akhter, Enamul Kabir, Mohammad Shamiul Islam

**Affiliations:** ^1^Uttara Adhunik Medical College, Dhaka, Bangladesh; ^2^Bangabandhu Sheikh Mujib Medical University, Dhaka, Bangladesh; ^3^Sir Salimullah Medical College, Dhaka, Bangladesh; ^4^Hospital Services Management, Directorate General of Health Services, Dhaka, Bangladesh

**Keywords:** Lupus nephritis, ISN/RPS classification, Systemic lupus erythematosus, Arthralgia

## Abstract

**Introduction:** The clinicopathological findings of lupus nephritis (LN) are responsible for the ultimate prognosis of systemic lupus erythematosus (SLE). But these findings show geographical variations. Data on LN of Bangladeshi patients are extremely rare in the literature and most of them describe mostly clinical features rather than the pathological findings.

**Objectives:** This study was carried out in an effort to find out the clinicopathological characteristics and correlations of LN patients from this region.

**Patients and Methods:** A total of 34 patients were included in the study; all these underwent renal biopsy. Each biopsy was classified according to International Society of Nephrology/Renal Pathology Society (ISN/RPS) 2003 LN classification system and compared with the clinical, biochemical and immunological findings.

**Results:** Arthralgia and edema were found to be the most common clinical presentations and both were present in 28 (82.4%) cases. Out of 34 cases, 22 (64.7%) belonged to ISN/RPS class IVG. Among clinical and biochemical findings, arthralgia and serum creatinine showed significant association with the ISN/RPS 2003 classification system of LN. The most common deposited immunoglobulin was IgG. This was present in 29 (85.29%) cases.

**Conclusion:** Observations made from the present study suggest that clinical and laboratory parameters of Bangladeshi patients do not predict the histological findings; though, some of the clinical and biochemical parameters correlated with histological findings in the present study. It was also found that these findings are different from other regional studies on LN.

**Implication for health policy/practice/research/medical education:**

Studies suggest that clinicopathological findings of lupus nephritis (LN) patients differ from region to region. Findings of Bangladeshi patients are rare in the literature and most of them are related to the clinical findings rather than pathological data; though pathological findings guide treatment protocol. Our study showed association of different clinical and pathological findings which could guide the clinicians to treat LN patients of this region.

## Introduction

Systemic lupus erythematosus (SLE) is a heterogeneous disease of disordered immunity, marked clinically by multi-systemic inflammatory involvement (1). Renal involvement is a frequent feature of SLE, occurring in 40%-75% in various populations of patients, most often within five years of the disease onset (2). It is one of the most significant causes of morbidity, health expenditure and mortality in patients with SLE (3). The clinical features of SLE have been extensively described from different geographical parts in the world, with some variations among different racial groups (4-6). The course of lupus nephritis (LN) is highly variable and multiple clinical, serological, histopathological and time dependent factors are responsible for its ultimate prognosis (7). However, data on LN of Bangladeshi patients are extremely rare in the literature and most of them describe mostly clinical features rather than pathological findings. So this study was carried out in an effort to find out the clinicopathological characteristics of LN patients from this region.

## Patients and Methods

### Study and settings

The study was carried out in the department of pathology, Sir Salimullah Medical College and Mitford Hospital, Dhaka during the period of July 2010 to June 2012. A total 34 patients were included in the study, who were diagnosed as symptomatic SLE on the basis of criteria of American College of Rheumatology and had undergone renal biopsy. Each renal biopsy was studied under light microscopy and immunofluorescent microscopy and was classified according to ISN/RPS 2003 LN classification system. For light microscopy, Hematoxylin and Eosin (H&E) stain, Periodic Acid-Schiff (PAS) stain and Masson’s trichrome stain were performed too. For immunofluorescent staining, each section was stained with fluorescein isothiocyanate (FITC)-conjugated rabbit anti-sera against human IgG, IgM, IgA, complement C3, C1q and fibrinogen (MEDIC, Torino, Italy). The sections were examined under immunofluorescence microscope (Hertel and Reuss, Germany, with exciter filter BP485 and barrier BP5200). The degree of fluorescence was graded on an arbitrary scale from (+) to (+++). For rest of the staining procedure, manufacturer’s instructions were followed. Control slides were stained with the same procedure.

### Diagnostic criteria of lupus nephritis

All those patients were included in the study who fulfilled the following criteria.

(*a*) Persistent proteinuria of greater than 0.5 g per day (or greater than 3+ urine dipstick reaction for albumin), or

(*b*) Cellular casts, including red blood cell, hemoglobin, granular, renal tubular cell, or mixed.

(*c*) And the biopsy containing at least 5 to 10 glomeruli according to pathologic findings (at least 5 glomeruli for diffuse LN and 8 to 10 for focal glomerulonephritis).

### Demographic, clinical and laboratory data

All the data were collected at the time of biopsy. These were age, sex, presence of malar rash, discoid rash, photosensitivity, arthralgia, oral ulcer, edema, neurologic signs, gross hematuria, blood pressure, serum creatinine, anti-dsDNA and UTP. To facilitate statistical analysis of serum creatinine, anti-dsDNA and UTP, patients were divided into two groups based on specific criteria.

### Ethical issues

The research followed the tenets of the Declaration of Helsinki. Informed written consent in Bangla was taken from each patient free of duress and without exploiting any weakness of subjects. In case of children, consent was taken from legal guardians. The research was approved by institutional ethical committee of Sir Salimullah Medical College and Hospital.

### Statistical analysis

Statistical analysis was done with statistical software SPSS for windows version 17 (SPSS, Chicago, IL, USA). Descriptive statistics such as mean ± standard deviation (SD) were used for continuous variables such as age and laboratory data. Numbers (%) were used to describe the proportion of categorical variables such as sex and the frequency of morphological variants. Statistical analysis was done using appropriate statistical tests, such as the chi-Square test and spearman rank correlation. A *P* value of ≤0.05 was considered to be significant.

## Results

A total of 34 cases of LN were included in the present study through convenience sampling technique. Mean age of the patients was 26±11.97 years and the age range was 10 to 60 years. Nine patients were children (<15 years). Out of 34 patients, 31 (91.18%) patients were females and only 3 (8.82%) patients were male.

The most common clinical presentations of the patients at the time of renal biopsy were arthralgia and edema. Both the symptoms were present in 82.4% of cases. The next frequent clinical presentation was malar rash which was present in 73.5% of patients. Neurologic manifestations were absent in all of the cases (Table 1).

**Table 1:** Clinical presentation of the patients of lupus nephritis at the time of renal biopsy

**Clinical presentations**	**Total**	**Percent**
Malar rash	25	73.5
Discoid rash	6	17.6
Photosensitivity	2	5.9
Arthralgia	28	82.4
Edema	28	82.4
Hypertension	8	23.5
Oral ulcer	13	38.2
Neurologic symptoms	0	0

Twenty-two (64.7%) patients belonged to ISN/RPS class IVG. None of the patients belonged to class I, class V and class VI (Table 2). Clinical presentations of the patients and ISN/RPS classification system were plotted in the Table 3. Chi-square test was performed. Only arthralgia showed significant association (*P*=0.011) among all the clinical symptoms.

**Table 2:** Frequency distribution of patients according to ISN/RPS classification of lupus nephritis

**ISN/RPS classification**	**Number**	**Percent**
Class I	0	0
Class II	4	11.8
Class III	4	11.8
Class IVG	22	64.7
Class IVS	4	11.8
Class V	0	0
Class VI	0	0

**Table 3:** Association between clinical presentation and ISN/RPS classification system

**Clinical Presentations**	**ISN/RPS II (n=4)**	**ISN/RPS III (n=4)**	**ISN/RPS IVG (n=22)**	**ISN/RPS IVS (n=4)**	**Total (n=34)**	***P*** ** value**
Malar rash	3	3	15	4	25	0.621
Discoid rash	0	1	15	0	6	0.521
Photosensitivity	0	0	1	1	2	0.360
Arthralgia	1	3	20	4	28	0.011*
Edema	2	3	19	4	28	0.248
Hypertension	0	0	8	0	8	0.127
Oral ulcer	3	2	7	1	13	0.361
Neurologic symptom	0	0	0	0	0	0

Chi-square test was done between biochemical parameters (anti-dsDNA, serum creatinine and urinary total protein [UTP]) and ISN/RPS classification system. All these parameters were divided into two categories as follows: anti-ds DNA level >100 IU/ml, serum creatinine level >1 mg/dl and UTP <1 mg/day were grouped in one group. Then these parameters were plotted in Table 4. Among these parameters, only serum creatinine showed highly significant association with ISN/RPS classification system of LN (*P*=0.001).

**Table 4:** Association between biochemical parameters and ISN/RPS classification system

**Biochemical Parameter**	**ISN/RPS II (n=4)**	**ISN/RPS III (n=4)**	**ISN/RPS IV-G (n=22)**	**ISN/RPS IV-S (n=4)**	**Total (n=34)**	***P*** **value**
Anti-dsDNA >100 IU/ml	4	4	20	4	32	0.763
S. Creatinine > 1 mg/dl	0	2	22	4	28	0.001*
UP >1 mg/day	4	2	18	3	27	0.351

Abbreviation: UP, urinary protein.

**Figure 1:**
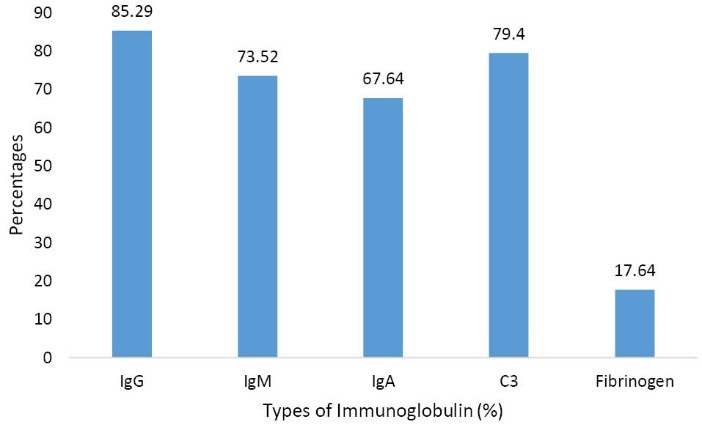
Distribution of immunoglobulins, complement and fibrinogen in renal biopsies.

Among immunoglobulin deposition, IgG was found to be the most frequent and it was present in 29 cases. In ISN/RPS, class IV-G showed deposition of IgG in 19 cases. C3 deposition was found in 27 cases. Fibrinogen deposition was found in 6 cases (Figure 1).

## Discussion

This present study was carried out to evaluate the clinicopathological characteristics of LN among Bangladeshi patients. Renal involvement is common in SLE (8). It is highly important to investigate whether signs of nephropathy are present or not in patients with SLE; since patients with renal disease have a poorer prognosis than those without kidney involvement (9). The clinical presentation of LN is highly variable, and the occurrence of kidney disease is the most important predictor of morbidity and mortality in patients with SLE (10).

The study included both adult patients and children. Mean age of the patients was 26±11.97 years. Another study from Bangabandhu Sheikh Mujib Medical University, Bangladesh, in 2006 showed mean age of the LN patients of 25.5±8.8 years (11). Similar studies were carried out in Singapore and China showing the mean age of the patient as 35.4 years and 33±14 years, respectively (12,13). A study in Iran showed mean age of 25.6±10.3 years and in other studies the mean age varied from 33.5±14 years to 36.8±13.8 years (14,15). However, the mean age of the patients of our country is concordant with the mean age of the patients of Iran but differs with the mean age of the patient of LN in China and Singapore. This supports the fact that our patients with LN were a decade younger than their Chinese counterparts indicating an earlier age of disease onset, more severe form of disease, or earlier mortality (16). Present study shows male: female ratio of 1:10 which is similar with a previous study from Iran with a male: female ratio of 1:13 (17). But present study differs with the study carried out in Singapore showing a male: female ratio of 1:4 (11). This difference could be due to racial and geographical variation of LN. This establishes the fact that clinical manifestations vary according to geographic location of the patients with LN (18,19).

In the present study, it is found that ISN/RPS class IVG as the most frequent, representing about 64.7% of the total cases and 11.8% cases were class IVS patients. In a cohort study carried out in China with 172 patients’ belonging to ISN/RPS class IV patients, they found 152 cases in class IVG and only 20 cases in class IVS (10).

In our study, only arthralgia (*P*=0.011) showed significant association with ISN/RPS classification system. Similar clinicopathological study was done by Nezhad et al (19). In their study, they found that edema (*P*=0.004) and hypertension (*P*=0.001) had significant association with ISN/RPS classification system but arthralgia did not show any significant association (19).

When statistical analyses were done between different biochemical parameters and ISN/RPS classification system, it was found that only serum creatinine showed significant association. Two other studies by Nezhad et al (19), and Yu et al (8) found similar observations. Perry et al (20) and Howie et al (21) also noticed serum creatinine as the test that showed significant correlation with Spearman’s rank correlation coefficient of 0.63 (*P*<0.01). Leaker et al did not find any correlation between the ANA and anti-dsDNA level with ISN/RPS classification system (22).

In present study, 100% cases showed glomerular deposits. The most common immunoglobulin found was IgG (85.29%). The next common was C3 which was present in 79.4% of renal biopsy specimens (Figure 1). Nossent et al found that 98.5% of their biopsies showed glomerular deposits and C3 was found to be most common (93%), followed by IgM (88%), IgA (84%) and IgG (78%) (23). Das et al also showed C3 deposition in 96.2% cases followed by IgM in 84.6% cases of patients of LN (24). They found full-house pattern of immunoglobulin deposition in 67% of the biopsies compared to 19 cases (59.3%) in the present study (24).

## Conclusion

Observations made from the present study suggest that clinical and laboratory parameters do not always predict the histological findings; though some of the clinical and biochemical parameters correlated with histological findings in the present study.

## Limitations of the study

Though essential, we could not study renal biopsy sample under electron microscope as facility for electron microscopy is not available in Bangladesh.

## Authors’ contribution

All authors contributed extensively to the work presented in this paper. SA checked the histopathological findings of the renal biopsy sample assisted in the technical aspects of the study. EK and MSI both guided the study and revised the manuscript equally.

## Conflicts of interest

The authors declared no competing interests.

## Ethical considerations

Ethical issues (including plagiarism, misconduct, data fabrication, falsification, double publication or submission, redundancy) have been completely observed by the authors.

## Funding/Support

This research did not receive any specific grant from any funding agency in the public, commercial or not for profit sector.
